# Anesthetic challenges of the first successful living-donor uterus transplantation in Latin America: a case report

**DOI:** 10.1016/j.clinsp.2026.100920

**Published:** 2026-03-25

**Authors:** Rui Carlos Detsch Junior, Joel Avancini Rocha Filho, Daniel Negrini Medeiros, Luiz Marcelo Sá Malbouisson, Rafael Soares Nunes Pinheiro, Rubens Macedo Arantes Junior, Daniel Reis Weisberg, Wellington Andraus, Mats Brännström, Dani Ejzenberg, José Maria Soares Junior, Edmund Chada Baracat, Maria José Carvalho Carmona, Marcelo Luis Abramides Torres

**Affiliations:** a Discipline of Anesthesiology, Departamento de Cirurgia, Hospital das Clínicas, Faculdade de Medicina, Universidade de São Paulo (HCFMUSP), São Paulo, SP, Brazil; b Digestive Organs Transplant Division, Departamento de Gastroenterologia, Hospital das Clínicas, Faculdade de Medicina, Universidade de São Paulo (HCFMUSP), São Paulo, SP, Brazil; c Division of Gynecology, Departamento de Obstetricía e Ginecologia, Hospital das Clínicas, Faculdade de Medicina, Universidade de São Paulo (HCFMUSP), São Paulo, SP, Brazil; d Department of Obstetrics and Gynecology, Sahlgrenska Academy, University of Gothenburg, Gothenburg, Sweden

**Keywords:** Uterus transplantation, Living donors, Mayer-Rokitansky-Küster-Hauser syndrome, Transplant anesthesia, Multidisciplinary approach

## Abstract

**Background:**

Uterus transplantation is an emerging treatment for absolute uterine factor infertility. While surgically complex, the procedure also presents unique challenges for anesthetic management, particularly in living-donor scenarios, where two major operations proceed in close succession with partial temporal overlap. The authors report the anesthetic management and challenges of the first successful living-donor uterus transplantation in Latin America.

**Case presentation:**

A 34-year-old female recipient with Mayer-Rokitansky-Küster-Hauser syndrome and her 31-year-old sister, with two previous vaginal births, as the living-donor. The donor's anesthesia was challenging due to the prolonged surgical time, requiring extended general anesthesia with extended mechanical ventilation and continuous invasive monitoring. Another key challenge was managing the donor's significant postoperative pain, necessitating a robust multimodal approach. The recipient's anesthetic course focused on maintaining hemodynamic stability, normothermia, and appropriate fluid balance during major fluid shifts, vascular clamping, and graft reperfusion. Both patients remained hemodynamically stable without vasopressor support throughout their respective procedures.

**Conclusion:**

This landmark case demonstrates that successful living-donor uterus transplantation is feasible in a new clinical setting but underscores the critical importance of a specialized, proactive anesthetic strategy. Anesthesiologists must be prepared for prolonged surgical times, control postoperative pain in the donor, and complex hemodynamic management in the recipient. A multidisciplinary approach, psychological support, and comprehensive strategies to minimize perioperative discomfort for the donor, are crucial for optimizing outcomes for both donor and recipient.

## Highlights

• First successful living-donor uterus transplantation in Latin America is reported.
• Proactive, multimodal pain control is crucial for the healthy living donor.
• Hemodynamic stability in the recipient is paramount during graft reperfusion.
• A proactive anesthetic strategy is a critical pillar for a successful outcome.

## Introduction

Absolute Uterine Factor Infertility (AUFI) is a previously untreatable form of female infertility caused by either the absence of a uterus (congenital or surgical) or the presence of a non-functional uterus.^1^ This condition affects an estimated 1 in 500 women of reproductive age globally.^1^ For these women, the only pathways to parenthood were historically limited to adoption or gestational surrogacy, options that may not be desired or accessible due to personal, legal, financial, or ethical reasons.^2^^,^^3^ Uterus Transplantation (UTx) has emerged as a revolutionary treatment, offering women with AUFI the unique opportunity to experience genetic, gestational, and legal motherhood, closely resembling the natural course of motherhood.^4^^,^^5^

The clinical feasibility of the procedure was established with the landmark first live birth in Sweden in 2014, which resulted from a Living Donor (LD) transplant.^1^ Subsequent milestones, including the first live birth from a Deceased Donor (DD) in Brazil in 2017, performed at our Institution, have further advanced the field.^1^ To date, more than 90 UTx procedures have been performed worldwide using both living and deceased donors, resulting in over 45 live births.^2^ This progress has catalyzed the transition of UTx from a purely experimental concept to a viable clinical reality for a select group of patients.^1^^,^^6^ The UTx procedure is now being introduced as a clinical procedure, with coverage of the national health insurance system, in Germany and in Sweden.

The success of this complex procedure relies on a highly coordinated multidisciplinary team, in which anesthetic management is a cornerstone. The anesthesiologist faces the unique challenge of ensuring the safety and comfort of a healthy donor through a major and prolonged surgery, while simultaneously maintaining precise hemodynamic stability and optimal graft perfusion in the recipient. This dual responsibility presents a series of specific perioperative challenges that are critical to the overall outcome of the transplant. Specifically for UTx, as compared to traditional solid organ transplants, such as the kidney and liver, there is a long time from transplantation until a transplantation procedure of a uterus can be deemed fully successful, by a live birth. Thus, in UTx, success rates are usually divided into two phases. The first phase is surgical success, which is generally defined as the presence of a uterus showing adequate blood flow and menstruation. This will indicate functionality. However, the ultimate success of a UTx procedure is when a healthy child is born after UTx, which typically takes place from the second year after transplantation surgery.

Herein, the authors report the first surgically successful living-donor uterus transplantation performed in Latin America, with a primary focus on the specific anesthetic challenges encountered and the management strategies employed for both the donor and the recipient. This case report was prepared following the CARE (Case Report) guidelines for reporting clinical cases.

## Case report

This report details the first successful living-donor uterine transplantation in Latin America, performed at a public university hospital in Brazil. Comprehensive written and oral informed consent was obtained from both the recipient and the donor for all surgical and anesthetic procedures, and for the publication of these findings. This study was approved by the Ethics Committee of Hospital das Clinicas da Faculdade de Medicina da Universidade de São Paulo under protocol number (CAAE) 94938425.0.0000.0068.

### Recipient profile and pre-transplant history

The recipient was a 34-year-old woman with a Body Mass Index (BMI) of 27.8 kg/m^2^, diagnosed at age 15 with congenital uterine absence due to Mayer-Rokitansky-Küster-Hauser (MRKH) syndrome. The diagnosis of primary amenorrhea was established following a standard diagnostic workup, which revealed a normal 46, XX karyotype and normal Follicle-Stimulating Hormone (FSH) levels, consistent with preserved ovarian function. Despite the absence of menstruation, the patient reported experiencing cyclical symptoms such as mastalgia, bloating, and headaches, indicative of normal hormonal cycles. Physical examination revealed a short, blind-ending vagina, and pelvic ultrasonography confirmed the absence of a uterus but the presence of ovaries. A subsequent pelvic angio-CT scan reaffirmed the absence of the uterine body and cervix, noting only thin arterial branches (0.2 cm caliber) from the internal iliac arteries in the expected uterine topography. A corpus luteum cyst was visible in the right ovary, and no renal anomalies were detected.

Her medical history was unremarkable, with no comorbidities, other congenital malformations, or prior abdominal surgeries, and she was classified as American Society of Anesthesiologists (ASA) Physical Status I. Preoperative cardiac evaluation with an Electrocardiogram (ECG) and transthoracic echocardiogram was normal.

To address her infertility, the patient had undergone standard controlled ovarian stimulation, leading to the cryopreservation of twelve blastocysts following In Vitro Fertilization (IVF) with her husband's semen.

### Donor profile

The donor was the recipient's 31-year-old sister, classified as ASA Physical Status I with a Body Mass Index (BMI) of 24 kg/m^2^. She had no comorbidities and was not on any regular medication. Her obstetric history included two successful full-term vaginal deliveries. At the time of uterus donation, the children were 6- and 4-year-old, and the donor had undergone extensive psychological counseling, confirming that she had fulfilled her wish for childbearing. She reported allergies to dipyrone, paracetamol, ibuprofen, dexamethasone, and amoxicillin. The donor met all stringent inclusion criteria, including being pre-menopausal with completed parity, having a history of at least one live birth, negative serologies for HIV, Hepatitis B, and C, and no history of major uterine surgery.

### Anesthetic management and surgical procedure: donor

The primary anesthetic goals for the donor, a healthy patient undergoing major surgery for altruistic reasons, were to ensure maximal safety, maintain hemodynamic stability to facilitate surgical dissection, maintain adequate blood flow of the uterus, minimize blood loss, and provide excellent postoperative analgesia. Given the donor's extensive and significant allergy profile (including dipyrone, paracetamol, ibuprofen, and dexamethasone) noted previously, the perioperative plan was carefully constructed. This limited options for both antimicrobial prophylaxis and multimodal analgesia, reinforcing the decision to rely on an opioid-based neuraxial and systemic strategy combined with non-traditional adjuncts like ketamine. The anesthetic strategy for the donor's open hysterectomy involved a combination of general anesthesia and neuraxial analgesia. Standard non-invasive monitoring was implemented. Following placement of a 20-G peripheral Intravenous (IV) line and administration of 3 mg of IV midazolam, spinal anesthesia was performed with a 27-gauge Whitacre needle, delivering 200 µg of morphine and 25 µg of fentanyl intrathecally.

Advanced hemodynamic monitoring was established for precise physiological assessment. This included a 20 G arterial cannula in the left radial artery for continuous invasive blood pressure and Stroke Volume Variation (SVV) monitoring, and an ultrasound-guided 7Fr triple-lumen central venous catheter in the right internal jugular vein. Goal-Directed Fluid Therapy (GDFT) was employed to optimize intravascular volume, with the target of maintaining Stroke Volume Variation (SVV) below 13%. Crystalloid boluses were administered in response to increases in SVV, ensuring adequate organ perfusion while avoiding fluid overload. A target Mean Arterial Pressure (MAP) of > 65 mmHg was maintained throughout the procedure. Neuromonitoring included Bispectral Index (BIS), with a target range of 40‒60, and cerebral oximetry.

Given the anticipated prolonged surgical duration, meticulous attention was paid to patient positioning and measures to prevent complications associated with immobility. Intermittent pneumatic compression devices were applied to the lower limbs for deep vein thrombosis prophylaxis. All bony prominences, including the occiput, sacrum, and heels, were protected with specialized pressure-relieving cushions. Furthermore, to mitigate the risk of pressure alopecia, the patient's head was carefully repositioned approximately every 45 min.

Anesthetic induction was performed using a rapid sequence technique with 150 mg of propofol, 40 µg of sufentanil, and 80 mg of succinylcholine, followed by orotracheal intubation facilitated by a videolaryngoscope. Anesthesia was maintained with 2% sevoflurane in a 50% oxygen/air mixture, supplemented by continuous IV infusions of remifentanil (0.1–0.3 µg/kg/min) and cisatracurium (3 µg/kg/min).

The hysterectomy was performed via an infraumbilical, midline laparotomy. Given the potential for significant hemorrhage during pelvic dissection, protocols for massive transfusion were available. Prophylactic tranexamic acid was administered, and an intraoperative blood salvage system (Cell Saver) was on standby, though its use was not necessary. Point-of-care coagulation monitoring with rotational thromboelastometry (ROTEM®) was available to guide coagulation management, but no abnormalities were detected. The procedure was technically uneventful and lasted 12 h, with an estimated blood loss of 320 mL. The donor remained hemodynamically stable throughout, requiring no vasopressor support. After explantation, the uterus was perfused on the back table with cold Custodiol® solution. The donor was successfully extubated in the operating room and transferred to the Intensive Care Unit (ICU).

### Anesthetic management and surgical procedure: recipient

The recipient was brought to the operating theater approximately 8 h following the donor's arrival in the operative suite, timed to coincide with the explantation of the uterus. The primary anesthetic goals for the recipient were to maintain hemodynamic stability during major fluid shifts and vascular clamping, optimize uterine graft perfusion post-reperfusion, and facilitate the safe administration of induction immunosuppressive therapy. The anesthetic management was identical to the donor's, combining general anesthesia with spinal analgesia, the same comprehensive multimodal monitoring, and the same rigorous protocols for patient positioning and protection against complications from prolonged surgery. This included the implementation of GDFT guided by SVV and a target MAP > 65 mmHg to ensure adequate perfusion pressure for the new graft.

The transplant was performed via an infraumbilical laparotomy. The pelvic bed was prepared with meticulous dissection of the bilateral external and internal iliac vessels and identification of the ureters. Vascular anastomoses were performed in an end-to-side fashion. On the right side, one uterine artery, one uterine vein, and one uterine-branch of the ovarian vein were anastomosed. On the left, one uterine artery and one uterine-branch of the ovarian vein were anastomosed. No neural anastomoses were performed. The graft was secured by creating an anastomosis between the allograft's vaginal cuff and the recipient's vaginal vault. The recorded ischemia times were cold ischemia 2 h and 24 min, and warm ischemia 1 h and 41 min, for a total ischemia time of 4 h and 5 min. The procedure was technically uneventful and lasted 6 h, with an estimated blood loss of 200 mL.

Particular attention was paid to the period immediately following vascular clamp release. No significant hemodynamic instability, such as post-reperfusion syndrome, was observed, and no acute vasopressor therapy was needed. Intraoperative Doppler ultrasonography immediately confirmed patent anastomoses with normal flow. The patient remained hemodynamically stable and was extubated in the operating room before transferring to the ICU.

### Immunosuppression and thromboprophylaxis

The immunosuppression regimen was initiated intraoperatively prior to graft reperfusion with 20 mg of basiliximab (an interleukin-2 receptor antagonist) and 500 mg of intravenous methylprednisolone. A second dose of basiliximab was scheduled for postoperative day 4. Maintenance therapy consisted of: Tacrolimus, titrated to achieve a target serum trough level of approximately 10 ng/mL; Azathioprine, 100 mg once daily; Methylprednisolone, started at 160 mg postoperatively and tapered by 40 mg daily. No adjustments to the protocol were necessary during hospitalization or after discharge. Immunosuppressant toxicity was monitored through periodic clinical and laboratory examinations.

For thromboprophylaxis, 5000 U of intravenous heparin was administered just before the release of vascular clamps and subsequently maintained on enoxaparin 40 mg SC twice daily until hospital discharge.

### Postoperative course

Both patients had an uneventful 2-day stay in the ICU without the need for vasoactive support. A central focus of the postoperative care was the intensive management of the donor's pain. Anticipating the significant surgical insult, her comfort was proactively managed by the specialized pain service. Upon admission to the ICU, this team built upon the intrathecal analgesia by initiating a continuous intravenous ketamine infusion and a fentanyl-based Patient-Controlled Analgesia (PCA) pump, which collectively provided effective comfort and facilitated early mobilization. Despite this, the donor recovered well functionally, ambulating on the first postoperative day.

Both patients were discharged simultaneously on the seventh postoperative day. Graft viability in the recipient was monitored with serial pelvic Doppler ultrasounds, which showed continued adequate perfusion.

## Discussion

This case represents a significant milestone as the first successful Living-Donor Uterus Transplantation (LD-UTx) in Latin America, establishing a new frontier for the treatment of Absolute Uterine Factor Infertility (AUFI) in the region. The successful outcome, marked by a viable graft and satisfactory postoperative recovery for both donor and recipient, demonstrates that this highly complex procedure can be safely implemented with a well-coordinated, multidisciplinary team within a public university hospital setting, a model consistent with the establishment of new international programs.^7^

The anesthetic and surgical management employed were critical to the success of this case. The donor hysterectomy was a lengthy procedure, spanning 12 h, which is consistent with early experiences from other centers using an open approach.^8^ While minimally invasive robotic techniques are being developed to reduce surgical time and donor morbidity,^9^ the open technique remains a validated approach. The comprehensive anesthetic management went beyond standard monitoring, incorporating a proactive strategy focused on hemodynamic optimization and preparedness for major complications. Specifically, the use of continuous invasive arterial pressure monitoring, beyond providing beat-to-beat hemodynamic data, was considered mandatory to avoid the potential for nerve injury and limb ischemia associated with frequent, repetitive non-invasive cuff cycling during such a prolonged procedure. The use of Goal-Directed Fluid Therapy (GDFT) guided by Stroke Volume Variation (SVV) was a cornerstone of this approach. This strategy is crucial in prolonged, major abdominal surgeries to mitigate the risks of both hypovolemia, which could compromise organ perfusion, and fluid overload, which is associated with increased postoperative complications.^20^

The authors’ proactive stance also included the availability of point-of-care coagulation monitoring with thromboelastometry and a blood salvage system, reflecting a high level of preparedness for coagulopathy and hemorrhage, even though significant bleeding did not occur.^20^ A key learning point from this case was the successful management of the donor's high-intensity postoperative pain, which was effectively controlled by the specialized pain service using a ketamine infusion and Patient-Controlled Analgesia (PCA). This finding underscores the significant surgical insult of donor hysterectomy and highlights the importance of a proactive, multimodal analgesic strategy, a critical consideration in the ethics and care of living donors.^10^

This experience directly aligns with the principles of Enhanced Recovery After Surgery (ERAS) pathways, which aim to reduce the physiological stress of surgery and promote faster functional recovery. Although a specific ERAS protocol for uterine donors is not yet standardized, the guidelines for major gynecologic surgery are highly applicable.^21^ Key components include preoperative counseling and nutritional optimization, avoidance of prolonged fasting, multimodal opioid-sparing analgesia (combining regional anesthesia with non-opioid systemic drugs), goal-directed fluid therapy, early mobilization, and early return to oral intake. The high analgesic requirements observed in the donor, despite neuraxial analgesia, reinforce the necessity of a formalized ERAS pathway with a robust, preemptive, and multimodal analgesic plan to address the high-intensity somatic and visceral pain inherent to this procedure.^21^

The recipient's diagnosis of Mayer-Rokitansky-Küster-Hauser (MRKH) syndrome is the most common indication for UTx worldwide, accounting for 94% of cases in the US experience.^11^ The use of a genetically related living donor is also a frequent practice, often involving mothers or sisters.^12^ The immunosuppression protocol, combining induction with basiliximab, and methylprednisolone followed by a maintenance regimen of tacrolimus, azathioprine, and prednisone, is consistent with protocols used in other successful UTx programs.^13^ The absence of acute rejection episodes or vascular complications, such as thrombosis (the leading cause of early graft loss)^14^ in the immediate postoperative period is a testament to the meticulous surgical technique and effective immunosuppressive management, aligning the positive outcomes with recent benchmarks from leading international centers.^15^ Furthermore, the recipient's stable hemodynamic course, particularly the absence of a significant post-reperfusion syndrome following graft revascularization, is noteworthy and speaks to effective anesthetic management in anticipating volume shifts and maintaining graft stability.^20^

The successful completion of this transplant in a public university hospital in Brazil is particularly noteworthy. It demonstrates that with adequate expertise, resources, and institutional support, this life-altering procedure can be made accessible outside the context of high-income private healthcare systems. This is especially relevant in Latin America, where gestational surrogacy faces significant legal and cultural barriers, making UTx a vital new option for women with AUFI.^3^^,^^16^ This achievement builds upon the own group's foundational experience, which reported the first deceased-donor uterine transplant in Latin America in 2016^17^ an experience that later culminated in the world’s first live birth from a deceased donor.^18^ The establishment of living-donor program follows the current best-practice guidelines for new centers, ensuring rigorous patient selection and multidisciplinary collaboration from the outset.^7^^,^^19^

While this report is limited to the successful outcome of a single case, it serves as a proof-of-concept and a foundational experience. It confirms that the complex logistical, surgical, and clinical challenges of living-donor uterus transplantation can be overcome in a new geographical and healthcare context. This achievement not only offers hope to women with AUFI in Latin America but also provides a valuable blueprint for other centers in the region aspiring to establish similar programs.

## Conclusion

This case report documents the first successful living-donor uterus transplantation in Latin America, a milestone achieved through intensive multidisciplinary collaboration. The success of this pioneering procedure was critically dependent not only on surgical expertise but also on a sophisticated and proactive anesthetic strategy tailored to the unique demands of both the donor and the recipient.

The authors’ experience underscores that advanced hemodynamic management, utilizing goal-directed fluid therapy, and a high level of preparedness for major complications are essential for maintaining patient stability through these prolonged and complex operations. Furthermore, it highlights that effective management of the donor's significant postoperative pain is a fundamental challenge that requires a robust, multimodal approach.

In conclusion, this case demonstrates that establishing a successful uterus transplantation program is feasible within a public university hospital setting in a new geographical region. More importantly, it provides a detailed blueprint of the essential anesthetic considerations, confirming that anesthetic preparedness and expertise are indispensable pillars for the safety and success of this life-altering procedure.

## Data availability statement

All data generated or analyzed during this study are included in this published article.

## Declaration of competing interest

The authors declare no conflicts of interest.
